# Inhibitory effects of p-coumaric acid against avian pathogenic *Escherichia coli* O2:K1 in vitro and in vivo

**DOI:** 10.1016/j.psj.2026.107413

**Published:** 2026-07-08

**Authors:** Yuxin Li, Baichang Xu, Yuxi Qin, Geyin Zhang, Jia Cao, Shiqi Luo, Jieyu Su, Yiming Li, Zhongyang Ma, Jiesheng Liao, Zhenqin Yan, Zhaojia Ding, Hongbin Si

**Affiliations:** College of Animal Science and Technology, Guangxi University Key Laboratory of Animal Breeding, Disease Control and Prevention, Guangxi grass station, Guangxi University, Nanning, 530004, Guangxi, China

**Keywords:** p-coumaric acid, Avian pathogenic *Escherichia coli*, Virulence genes, Antibacterial activity, Inflammatory cytokines

## Abstract

Avian colibacillosis, caused by avian pathogenic *Escherichia coli* (APEC), is a severe bacterial disease in the poultry industry. With the increasing problem of antibiotic resistance, safe and effective alternatives are urgently needed. p-Coumaric acid (pCA) is a natural phenolic compound with reported antimicrobial activity; however, systematic data regarding its effects against APEC O2:K1 both *in vitro* and *in vivo* remain limited. In this study, the minimum inhibitory concentration (MIC) of pCA against APEC O2:K1 was determined by the broth microdilution method. The *in vitro* antibacterial activity was evaluated by growth curve analysis, crystal violet staining, drug-containing plate diffusion assay, nucleic acid and protein leakage tests, and the effect on virulence gene expression was examined by quantitative real-time PCR (qPCR). An infection model was established by intraperitoneal injection of APEC O2:K1 into 14-day-old chicks. The chicks were randomly divided into blank control group, model group, low, medium, and high-dose pCA groups (50, 100, and 200 mg/kg), and enrofloxacin positive control group (100 mg/kg). Chicks received the respective treatments via oral gavage once daily for three consecutive days post-challenge. Survival rate was observed for seven days, and organ indices, bacterial loads, serum inflammatory cytokines, and oxidative stress markers were measured. Heart and liver tissues were collected for histopathological examination. The MIC of pCA against APEC O2:K1 was 3.125 mg/mL. pCA inhibited bacterial growth in a concentration-dependent manner. At 1/2 MIC, the inhibition rates of biofilm formation and colony spreading were 71.4% and 47.6%, respectively. pCA significantly disrupted bacterial cell membrane integrity, leading to leakage of intracellular nucleic acids and proteins. At sub-inhibitory concentrations, pCA significantly down-regulated the expression of virulence genes, including *luxS, fimH, ompA, iss, iutA, tsh*, and *ibeA*. In the *in vivo* experiment, the seven-day survival rate of the model group was 45.83%, whereas that of the medium-dose pCA group (100 mg/kg) was 83.33% (*P* = 0.007). Compared with the model group, the medium-dose pCA group showed significantly reduced heart, liver, lung, and spleen indices, and recovered thymus and bursa of Fabricius indices. Bacterial loads in blood and liver were significantly decreased. Serum levels of pro-inflammatory cytokines (TNF-α, IL-1β, and IL-6), endotoxin LPS, and infection marker PCT were significantly reduced, while the anti-inflammatory cytokine IL-10 level was elevated. Immunoglobulin IgY and IgM levels returned to near normal ranges. The activities of antioxidant enzymes SOD, CAT, and GSH-Px in serum and liver were significantly increased, and the lipid peroxidation product MDA content was significantly decreased. Histopathological examination confirmed that pericarditis and perihepatitis lesions were markedly alleviated in the pCA treatment group. These results indicate that p-coumaric acid exerts significant therapeutic effects against avian colibacillosis through direct antibacterial activity and regulation of host inflammatory and oxidative stress responses, demonstrating its potential for development as a novel anti-APEC agent.

## Introduction

Avian colibacillosis, caused by avian pathogenic *Escherichia coli* (APEC), is a severe bacterial infectious disease characterized clinically by acute septicemia, fibrinous pericarditis, and perihepatitis (Watts and Wigley, 2024). Under intensive farming conditions, this disease causes high morbidity and mortality, resulting in substantial economic losses to the poultry industry (Saeed et al., 2025). Antibiotics have long been the primary means of controlling this disease. However, due to the complex serotypes and diverse virulence factors of APEC, coupled with improper use of antibiotics, multidrug-resistant strains have emerged continuously, leading to a decline in clinical treatment efficacy (Liu et al., 2024). Therefore, the development of safe, effective, and resistance-resistant alternative drugs has become an urgent requirement. p-Coumaric acid (pCA), also known as p-hydroxycinnamic acid, is a natural phenolic acid compound widely present in fruits, vegetables, cereals, and various medicinal plants (Silva et al., 2023). It possesses multiple biological activities, including antibacterial, anti-inflammatory, and antioxidant properties (Zhang et al., 2025). Studies have shown that pCA inhibits common *E. coli* by disrupting bacterial cell membranes and interfering with DNA synthesis (Lou et al., 2012), and enhances the bactericidal effect of antibiotics by blocking the bacterial SOS response (Ojha and Patil, 2019). In addition, pCA exhibits favorable pharmacological activities in anti-inflammatory and antioxidant aspects (Park et al., 2017; Yuan et al., 2023).

Although the above studies have revealed some biological functions of pCA, systematic reports on its antibacterial activity and *in vivo* therapeutic efficacy against the highly pathogenic avian APEC O2:K1 strain are still lacking. Data on its effect on the expression of key virulence factors of APEC also remain unclear.

APEC pathogenicity is mediated by multiple virulence determinants that coordinate bacterial adhesion, invasion, immune evasion, and iron acquisition. Adhesion to host epithelial cells is primarily mediated by type 1 fimbriae, whose tip adhesin FimH binds to mannosylated receptors on the host cell surface, representing a critical initial step of infection (Pourhossein et al., 2022). The outer membrane protein OmpA facilitates bacterial attachment and invasion, and its expression has been associated with the induction of host cell apoptosis during APEC infection (Afayibo et al., 2022). IbeA contributes to the ability of APEC to invade host cells; recent genomic surveys have confirmed that invasion-related genes including ibeB are highly prevalent among APEC isolates from diseased poultry (Afayibo et al., 2022).

Once established in the host, APEC employs Iss (increased serum survival) to resist complement-mediated killing; the iss gene is among the most frequently detected virulence markers in APEC, with prevalence exceeding 80% in multiple studies (Chen et al., 2021; Afayibo et al., 2022). For iron acquisition, APEC relies on the aerobactin uptake system encoded by the iutA gene, which has been identified as one of the predominant virulence-associated genes in APEC isolates (Goudarztalejerdi et al., 2020; Pourhossein et al., 2022). The temperature-sensitive hemagglutinin Tsh, a member of the autotransporter protein family, possesses both agglutinin and protease activities that facilitate colonization, and its prevalence among APEC strains has been reported to exceed 60% (Rajkhowa et al., 2020). Additionally, LuxS serves as a key regulator of the quorum sensing system; deletion of the luxS gene has been shown to significantly reduce bacterial adherence, invasion, motility, and overall virulence in APEC (Han et al., 2013). Collectively, these virulence factors enable APEC to establish systemic infection and cause severe disease in poultry.

O2:K1 is a common highly pathogenic serotype of APEC, and its K1 capsule and multiple virulence factors confer strong immune evasion and tissue invasion capabilities (Sarowska et al., 2022; Arredondo-Alonso et al., 2023). This study systematically evaluates the inhibitory effects of pCA against APEC O2:K1 both *in vitro* and *in vivo*, and analyzes its mechanism of action from three aspects—direct antibacterial activity, anti-virulence effects, and host immune regulation—with the aim of providing experimental evidence for the development of new drugs against avian colibacillosis.

## Materials and methods

### Bacterial strain and reagents

The APEC O2:K1 strain TW-XM was kindly provided by Professor Guoqiang Zhu (Yangzhou University, Yangzhou, China). p-Coumaric acid (purity ≥ 98%) was purchased from Shanghai Yuanye Bio-Technology Co., Ltd. LB broth, MHB broth, and MacConkey agar medium were purchased from Beijing Aoboxing Bio-Technology Co., Ltd. Chicken TNF-α, IL-1β, IL-6, IL-10, LPS, PCT, IgY, and IgM ELISA kits were purchased from Jiangsu Jingmei Biotechnology Co., Ltd. Superoxide dismutase (SOD), catalase (CAT), glutathione peroxidase (GSH-Px), and malondialdehyde (MDA) assay kits were purchased from Jiangsu Aidisheng Biotechnology Co., Ltd. TRIzol, reverse transcription kit, and SYBR qPCR Mix were purchased from Beijing GenStar Biotechnology Co., Ltd. Ten percent enrofloxacin soluble powder was purchased from Weifang Like Biotechnology Co., Ltd. Sodium carboxymethyl cellulose (CMC—Na) was purchased from Sinopharm Chemical Reagent Co., Ltd.

### Determination of minimum inhibitory concentration and growth curve

The minimum inhibitory concentration (MIC) of pCA against APEC O2:K1 was determined by the broth microdilution method according to Clinical and Laboratory Standards Institute (CLSI) guidelines. pCA was dissolved in dimethyl sulfoxide (DMSO) to prepare a 100 mg/mL stock solution and serially diluted two-fold with MHB broth in a 96-well plate. An equal volume of logarithmic phase bacterial culture (final concentration approximately 1 × 10⁶ CFU/mL) was added to each well. The plate was incubated at 37°C for 16–18 h, and the results were observed visually. The lowest drug concentration showing no visible bacterial growth was determined as the MIC. The experiment was repeated independently three times. For growth curve determination, fresh LB broth containing different final concentrations of pCA (MIC, 1/2 MIC, 1/4 MIC, 1/8 MIC) was inoculated with 1% of an overnight bacterial culture. The mixture was dispensed into a 96-well plate (200 μL per well) with three replicates per concentration. Control wells without drug and wells containing DMSO (solvent control) were also included. The plate was incubated in a microplate reader at 37°C with shaking for 18 h, and OD600 values were measured every 30 min. The growth curve was plotted with time on the x-axis and OD600 values on the y-axis. The experiment was repeated independently three times.

### Biofilm formation inhibition assay

The effect of pCA on APEC biofilm formation was evaluated by crystal violet staining (Peng et al., 2018). Bacterial suspension at a concentration of 1 × 10⁶ CFU/mL was added to a 96-well plate (200 μL per well). pCA was added to final concentrations of 1/2 MIC, 1/4 MIC, and 1/8 MIC, with three replicate wells per concentration. An untreated positive control group and a blank medium control group were also included. After static incubation at 37°C for 48 h, the supernatant was discarded, and the wells were gently washed three times with sterile PBS. Each well was fixed with 200 μL of methanol for 20 min, after which the methanol was discarded and the plate was air-dried. Then, 200 μL of 0.1% crystal violet solution was added to each well for staining at room temperature for 15 min. The dye solution was discarded, and the wells were rinsed with running water until colorless and air-dried. Bound crystal violet was dissolved with 200 μL of 33% glacial acetic acid, and OD570 values were measured. For calculation of the inhibition rate, the OD570 value of the blank medium control group was first subtracted from the OD570 values of the untreated control group and each treatment group to obtain corrected OD values. The inhibition rate was then calculated using the following formula: Inhibition rate (%) = [(corrected OD value of untreated control group–corrected OD value of treatment group) / corrected OD value of untreated control group] × 100%.

### Drug-containing plate diffusion inhibition assay

Bacterial motility was assessed using a soft agar plate assay as described previously (Ye et al., 2022) with slight modifications. Mueller-Hinton Agar (MHA) plates containing different concentrations of pCA (1/8 MIC, 1/4 MIC, 1/2 MIC, 1 MIC) were prepared. Two microliters of logarithmic phase bacterial culture were spotted onto the center of each plate. After incubation at 37°C for 16–18 h, the diameter of the bacterial growth zone was measured using a vernier caliper in two perpendicular directions; plates without drug were used as controls. The inhibition rate of bacterial growth zone diameter was calculated. The experiment was repeated independently three times.

### Cell membrane permeability assay

Cell membrane permeability was assessed by measuring the leakage of nucleic acids and proteins as described previously (Gao et al., 2023) with slight modifications. An overnight bacterial culture was inoculated into fresh LB broth at a 1:100 ratio and cultured at 37°C with shaking at 220 rpm until mid-logarithmic phase (OD600 ≈ 0.5). Bacterial cells were collected by centrifugation at 4°C and 5,000 rpm for 10 min, washed twice with sterile PBS, and resuspended. The bacterial suspension was dispensed into sterile tubes, and pCA was added to final concentrations of 1/2 MIC, 1/4 MIC, and 1/8 MIC. Tubes without drug served as controls. After incubation at 37°C with shaking at 220 rpm for 2 h, the cultures were centrifuged at 4°C and 12,000 rpm for 5 min. The supernatant was collected, and nucleic acid and protein concentrations were measured using a microvolume spectrophotometer. Samples exceeding the detection limit were diluted 10‑fold with PBS prior to measurement. Three replicates were performed for each concentration.

### Virulence gene expression detection

Total RNA was extracted and subjected to qPCR analysis as described previously (Peng et al., 2018; Dong et al., 2019) with slight modifications. APEC cultures at mid-logarithmic phase (OD600 ≈ 0.5) were dispensed into sterile tubes, and pCA was added to final concentrations of 1/8 MIC, 1/4 MIC, and 1/2 MIC. After further incubation at 37°C with shaking at 220 rpm for 2 h, bacterial cells were collected by centrifugation at 4°C and 10,000 rpm for 5 min and washed twice with PBS. Total RNA was extracted using TRIzol reagent and reverse transcribed into cDNA. Using *16S rRNA* as an internal reference, the mRNA expression levels of *fimH, ompA, iss, iutA, tsh, luxS, ibeA, lpxC*, and *pgsA* were detected by qPCR. The reaction conditions were as follows: initial denaturation at 95°C for 2 min; 40 cycles of denaturation at 95°C for 15 s and annealing/extension at 60°C for 30 s. Relative expression levels were calculated using the 2⁻^ΔΔCt^ method. Primer sequences are listed in Table 1.

**Table 1:** Primer sequences used for quantitative real-time PCR.

Gene	Forward primer (5′→3′)	Reverse primer (5′→3′)
*16S rRNA*	TGGCGCATACAAAGAGAGC	ACTCCAATCCGGAGGACTACGAC
*luxS*	GATTGGTACGCCAGATGAGC	CGAGTGCATCTGGTAAGTGC
*fimH*	TCAGTGCCGATTCCTCTTAC	TTAATCCCAGACTTACCGCC
*iutA*	AGAAATTTGGCGGCTGGTTT	TTCAGCGTACCAGTTCCCAT
*ompA*	TGGGTGTTTCCTACCGTTTC	GAGTGAAGTGCTTGGTCTGT
*tsh*	CCACAGGATCGGGAAAGTTTAT	TTGCGAGCTGTAGCGTATTC
*ibeA*	CGCGATATGTTTAGCCCTTATCT	CCGCCTAACGTCACATCTTT
*iss*	CCGCTCTGGCAATGCTTATT	TTGTCCAATTCCCGAAACGA
*pgsA*	GTTTACCCAACTCCGCCATC	ACAGCTGTGTGGGTGACATTA
*lpxC*	ATCTATCGTCGCACCGACTT	TCGTTGACCAGACACGTACA

### Experimental animals and grouping

One-day-old Guangxi Jinling Hua chickens were purchased from Guangxi Jinling Agriculture and Animal Husbandry Group and housed at the Animal Experiment Center of Guangxi University under three-dimensional cage rearing conditions. Relative humidity was maintained at 55–65%, and temperature was controlled at 32–34°C for chicks aged 1–7 days, then reduced by 2–3°C weekly until reaching 26°C. The chicks had free access to antibiotic-free basal diet and water. The basal diet was formulated to meet the nutrient requirements for growing chicks, consisting of a corn-soybean meal-based mash with the following guaranteed analytical values: crude protein 16.0–18.0%, crude fiber ≤ 13.0%, crude ash ≤ 13.0%, calcium 0.6–1.50%, total phosphorus ≥ 0.3%, and sodium chloride 0.30–0.80%. A preliminary experiment with a vehicle control group (0.5% CMC—Na) showed no significant differences in any indices compared with the blank control group; therefore, no separate vehicle control was included in the formal experiment. The median lethal dose (LD_50_) of APEC O2:K1 by intraperitoneal injection was determined to be approximately 2.92 × 10⁷ CFU per chick using the modified Kärber method in a preliminary experiment.

A total of 144 healthy 14-day-old chicks, with body weights ranging within ±5 g, were randomly divided into six groups with 24 chicks per group. During the experimental period, all treatments were coded, and the code was not broken until data analysis was completed. For histopathological evaluation, tissue sections were examined blindly by two independent pathologists who were unaware of the group allocations. Group C (blank control): intraperitoneal injection of sterile saline, oral gavage with 1 mL drinking water. Group M (model): intraperitoneal injection of bacterial suspension, oral gavage with 1 mL 0.5% CMC—Na. Group pCA-L: intraperitoneal injection of bacterial suspension, oral gavage with 1 mL pCA at 50 mg/kg. Group pCA-M: intraperitoneal injection of bacterial suspension, oral gavage with 1 mL pCA at 100 mg/kg. Group pCA-H: intraperitoneal injection of bacterial suspension, oral gavage with 1 mL pCA at 200 mg/kg. Group PC (enrofloxacin positive control): intraperitoneal injection of bacterial suspension, oral gavage with 1 mL 10% enrofloxacin soluble powder at 100 mg/kg. Oral gavage was initiated 1 h after challenge and administered once daily for three consecutive days.

### Sample collection and index measurement

The chicks were observed continuously for seven days, and mortality was recorded. On day 7, surviving chicks were euthanized and necropsied. Gross lesions of the heart and liver were observed and photographed. The chicks were weighed, and the heart, liver, lungs, spleen, thymus, and bursa of Fabricius were dissected and weighed. Organ index (mg/g) was calculated as organ weight (mg) / body weight (g). Anticoagulated blood was collected aseptically, serially diluted, and plated on MacConkey agar. After incubation at 37°C for 18–24 h, bacterial colonies were counted to determine blood bacterial load (CFU/mL). Approximately 100 mg of liver tissue was aseptically collected and homogenized in 1 mL sterile PBS. The homogenate was serially diluted, plated, and counted to determine liver bacterial load (CFU/g). Serum levels of inflammatory cytokines (TNF-α, IL-1β, IL-6, IL-10), infection-related markers (LPS, PCT), and immunoglobulins (IgY, IgM) were measured using commercial ELISA kits. The activities of SOD, CAT, and GSH-Px and the MDA content in serum and liver homogenates were measured using biochemical assay kits. All procedures were performed strictly according to the kit instructions. Heart and liver tissues were fixed in 4% paraformaldehyde, dehydrated in graded ethanol, cleared in xylene, embedded in paraffin, sectioned at 4–5 μm thickness, stained with hematoxylin and eosin (HE), and examined under a light microscope.

For histopathological evaluation, a semi-quantitative scoring system was adopted based on previously described methods (Landmann et al., 2021). Heart and liver sections from each chick (n = 6 per group) were examined blindly by two independent pathologists. The severity of lesions, including inflammatory cell infiltration, oedema, necrosis, and structural disorganisation, was graded on a four-point scale: 0 = no significant lesions; 1 = mild (focal, < 25% of the field affected); 2 = moderate (multifocal, 25 - 50% affected); 3 = severe (diffuse, > 50% affected).

### Statistical analysis

All data were statistically analyzed using GraphPad Prism 8.0 software. Quantitative data are expressed as mean ± standard deviation (Mean ± SD). Comparisons among multiple groups were performed by one-way analysis of variance (One-way ANOVA), and pairwise comparisons between groups were performed by Tukey's multiple comparison test. Survival curves were plotted using the Kaplan-Meier method, and group comparisons were performed using the Log-rank test. *P* < 0.05 was considered statistically significant.

### Ethical approval

This study was approved by the Animal Care and Welfare Committee of Guangxi University (Approval No. GXU-2026-192), All efforts were made to minimize animal suffering.

## Results

### Effect of pCA on the MIC and growth curve of APEC

**Fig. 1:**
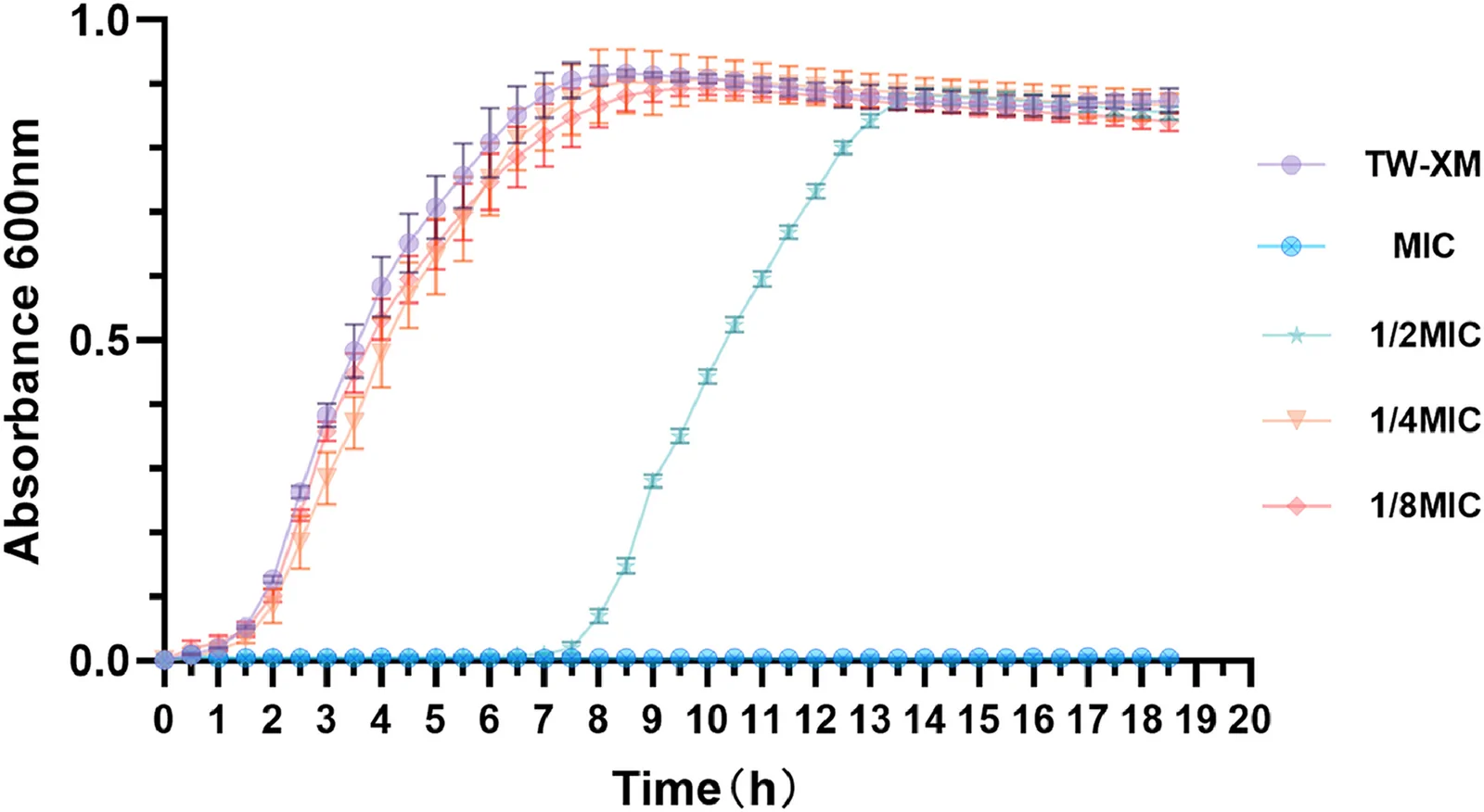
Effect of pCA on the growth curve of APEC O2:K1. APEC O2:K1 was cultured in LB broth containing different concentrations of pCA (MIC, 1/2 MIC, 1/4 MIC, 1/8 MIC) at 37°C for 18 h. OD600 values were measured every 30 min. Data are presented as mean ± SD (n = 3).

The broth microdilution assay showed that the MIC of pCA against APEC O2:K1 was 3.125 mg/mL. The growth curve (Fig. 1) showed that bacteria in the control group exhibited typical sigmoidal growth, whereas bacterial proliferation was completely inhibited in the MIC pCA treatment group. In the 1/2 MIC group, growth was arrested within the first 7 h of culture, followed by a slow recovery trend. The 1/4 MIC and 1/8 MIC groups showed relatively weak inhibition of bacterial growth. These results indicate that pCA inhibits the proliferation of APEC O2:K1 in a concentration-dependent manner.

### Inhibitory effect of pCA on APEC biofilm formation

**Fig. 2:**
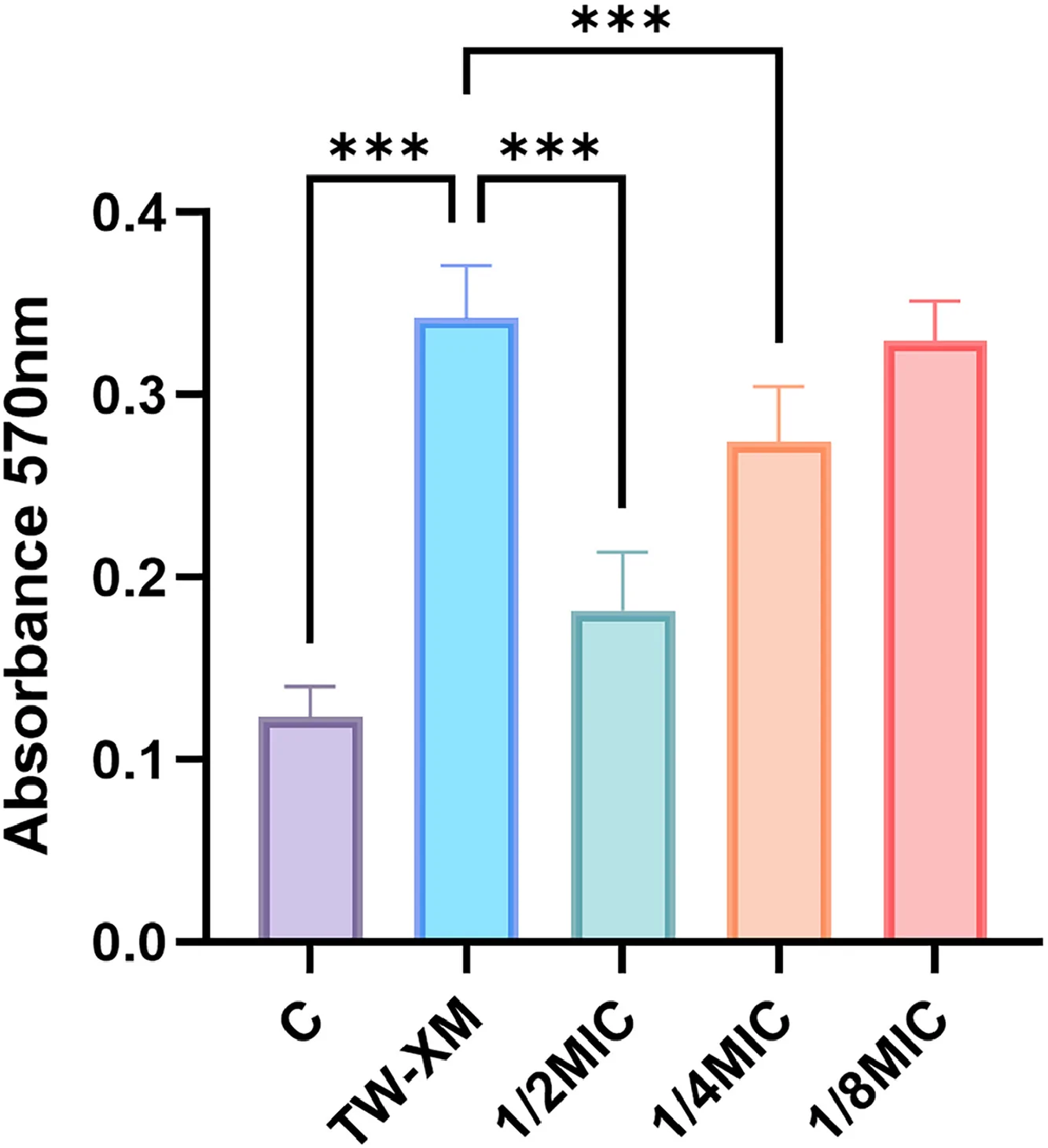
Inhibitory effect of pCA on biofilm formation of APEC O2:K1 Biofilm formation was quantified by crystal violet staining after 48 h treatment. C: blank medium control (MHB broth); TW-XM: untreated control; 1/8 MIC, 1/4 MIC, 1/2 MIC: pCA-treated groups. Data are presented as mean ± SD (n = 3). *P < 0.05, ^⁎⁎^*P* < 0.01, ^⁎⁎⁎^*P* < 0.001 vs. TW-XM.

The quantitative results of crystal violet staining (Fig. 2) showed that compared with the untreated control group, biofilm formation of APEC was significantly inhibited after treatment with 1/2 MIC, 1/4 MIC, and 1/8 MIC of pCA in a dose-dependent manner. The inhibition rates at 1/2 MIC, 1/4 MIC, and 1/8 MIC were 71.4% (*P* < 0.01), 31.3% (*P* < 0.05), and 6.6%, respectively. These results indicate that pCA effectively inhibits biofilm formation of APEC.

### Effect of pCA on APEC spreading ability

**Fig. 3:**
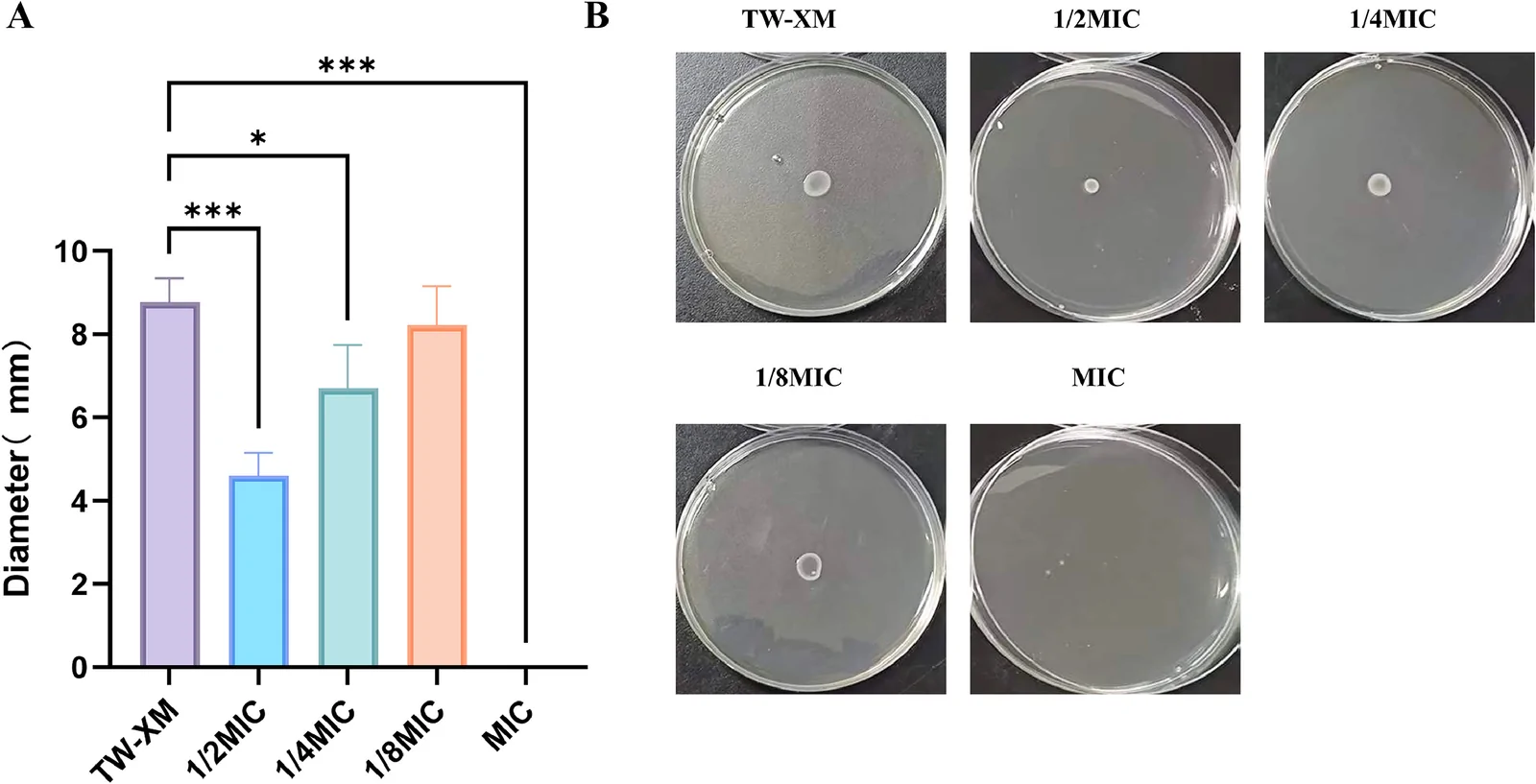
Inhibitory effect of pCA on the spreading of APEC O2:K1 on solid medium. (A) Representative images of colony spreading on MHA plates containing different concentrations of pCA (MIC, 1/2 MIC, 1/4 MIC, 1/8 MIC). (B) Quantitative analysis of colony diameter. Data in panel B are presented as mean ± SD (n = 3). *P < 0.05, ^⁎⁎^*P* < 0.01, ^⁎⁎⁎^*P* < 0.001 vs. untreated control.

The results of the drug-containing plate diffusion assay (Fig. 3A) showed that on MHA plates without drug, APEC exhibited obvious spreading growth, forming large colonies. As the concentration of pCA in the plates increased, the colony spreading area decreased significantly, with clear edges, and growth was completely inhibited at the MIC concentration. Quantitative measurement of colony diameter (Fig. 3B) showed that compared with the control group, the inhibition rates of colony diameter at 1/2 MIC, 1/4 MIC, and 1/8 MIC were 47.6% (*P* < 0.01), 23.6% (*P* < 0.05), and 6.2%, respectively. These results indicate that pCA effectively inhibits the colonization and spreading ability of APEC on solid medium surfaces.

### Effect of pCA on APEC cell membrane integrity

**Fig. 4:**
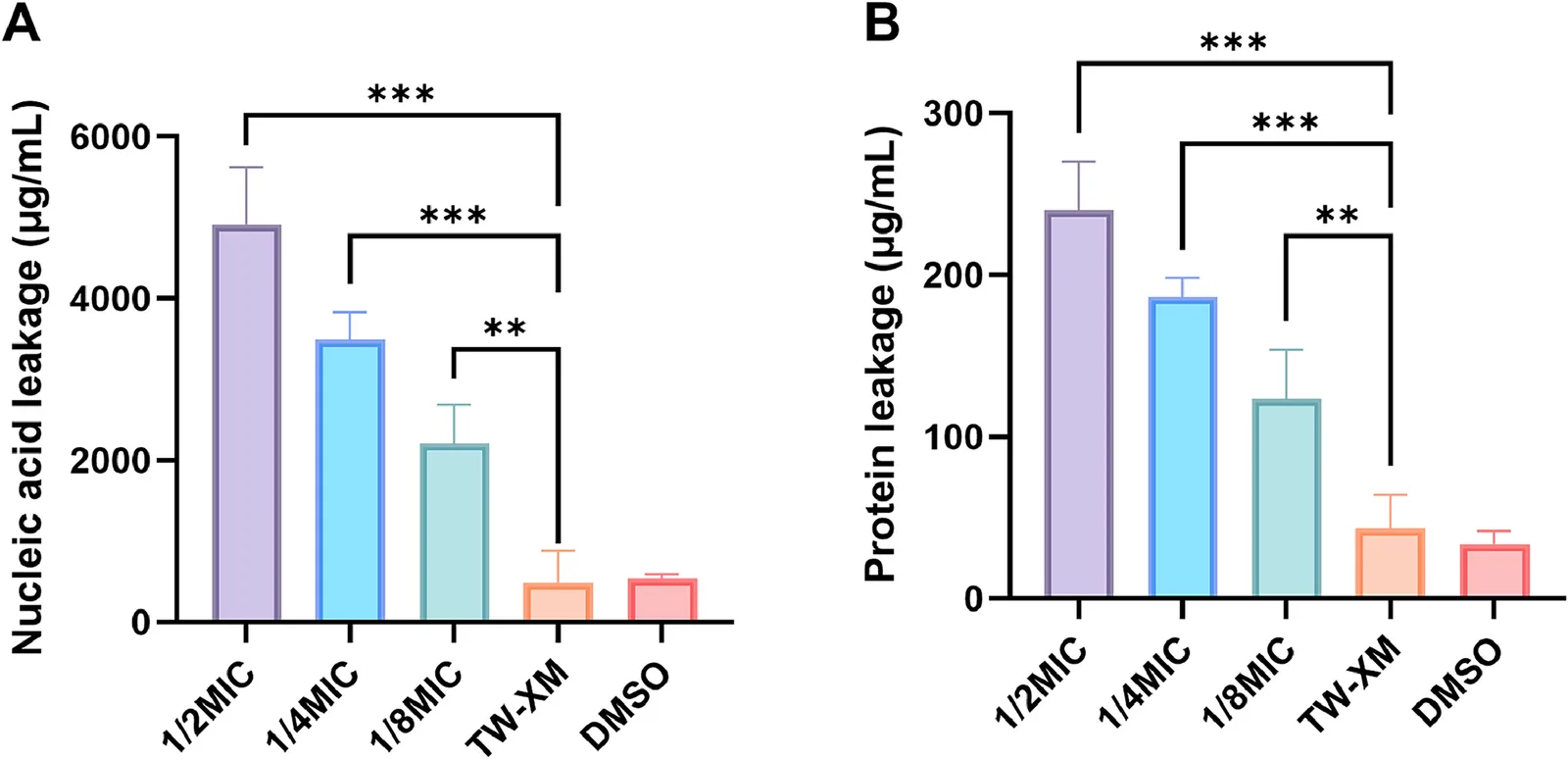
Effect of pCA on membrane permeability of APEC O2:K1. (A) Nucleic acid leakage in culture supernatant after treatment with different concentrations of pCA (1/2 MIC, 1/4 MIC, 1/8 MIC) for 2 h. (B) Protein leakage in culture supernatant after treatment with different concentrations of pCA for 2 h. Data are presented as mean ± SD (*n* = 3). *P < 0.05, ^⁎⁎^*P* < 0.01, ^⁎⁎⁎^*P* < 0.001 vs. untreated control.

The results of nucleic acid and protein leakage assays (Fig. 4) showed that compared with the untreated TW-XM group, the nucleic acid concentration in the supernatant was significantly increased after treatment with different concentrations of pCA (1/2 MIC, 1/4 MIC, 1/8 MIC) (*P* < 0.01 or *P* < 0.001), showing a concentration-dependent effect, with the highest leakage observed in the 1/2 MIC treatment group. The protein leakage results were highly consistent with the nucleic acid leakage results. This indicates that pCA directly damages the bacterial cell membrane structure and increases its permeability, leading to leakage of intracellular macromolecules.

### Effect of pCA on virulence gene expression in APEC

**Fig. 5:**
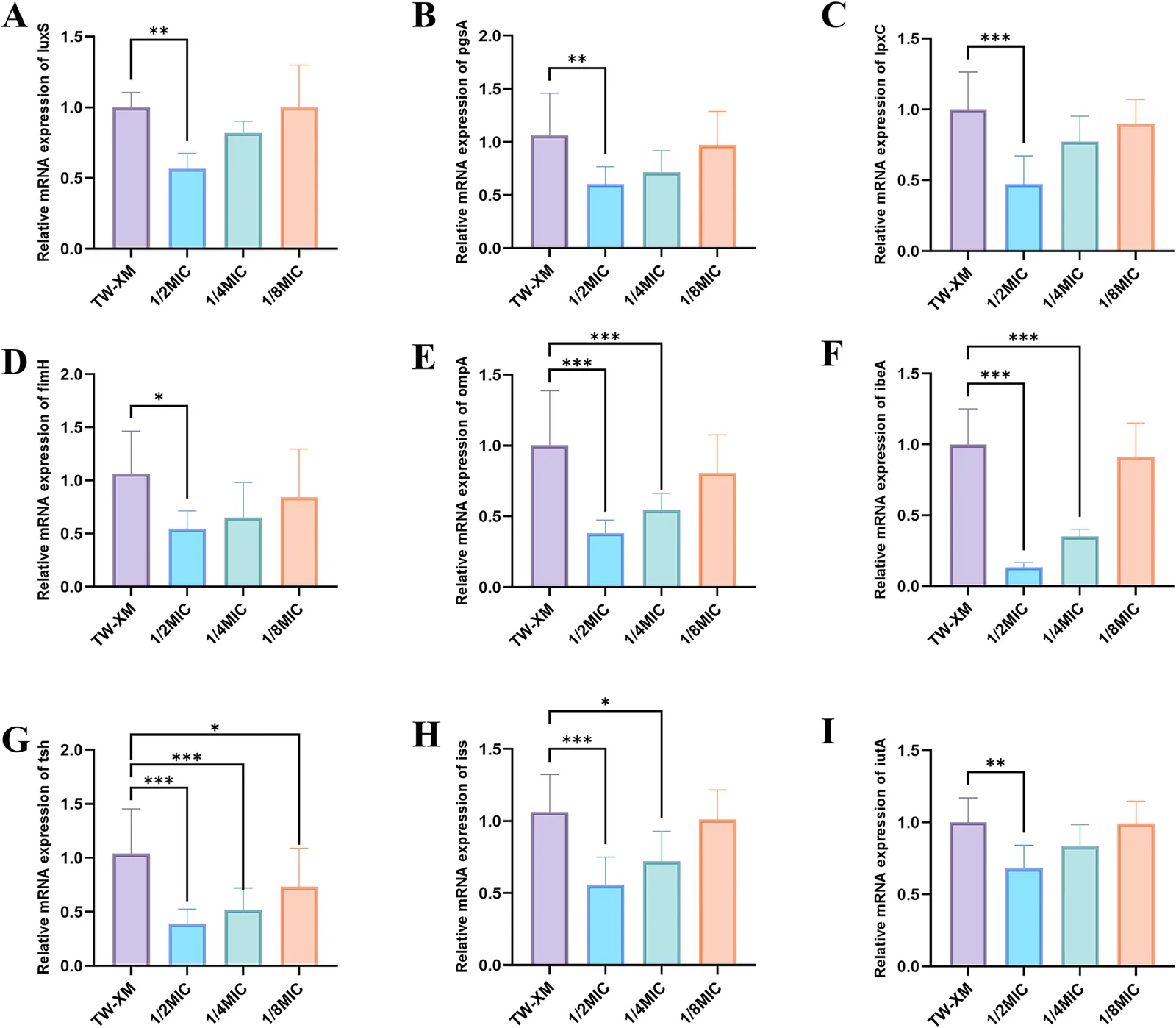
Effect of pCA on mRNA expression of virulence genes in APEC O2:K1. Panels show relative expression of: (A) *luxS*, (B) *pgsA*, (C) *lpxC*, (D) *fimH*, (E) *ompA*, (F) *ibeA*, (G) *tsh*, (H) *iss*, and (I) *iutA*. Data are presented as mean ± SD (*n* = 3). *P < 0.05, ^⁎⁎^*P* < 0.01, ^⁎⁎⁎^*P* < 0.001 vs. untreated control.

The qPCR results (Fig. 5) showed that pCA significantly down-regulated the transcription levels of the tested virulence genes in a dose-dependent manner. At sub-inhibitory concentrations, the expression of most genes was significantly inhibited (*P* < 0.001). Among them, the expression of *luxS*, a key regulatory gene of quorum sensing, was significantly suppressed. The expression of adhesion-related genes (*fimH, tsh*), invasion-related genes (*ompA, ibeA*), iron uptake gene (*iutA*), immune evasion gene (*iss*), and virulence factor synthesis-related genes (*lpxC, pgsA*) all decreased progressively with increasing drug concentration. This indicates that pCA at sub-inhibitory concentrations can systematically interfere with the virulence regulatory network of APEC.

### Effect of pCA on survival rate of infected chicks

**Fig. 6:**
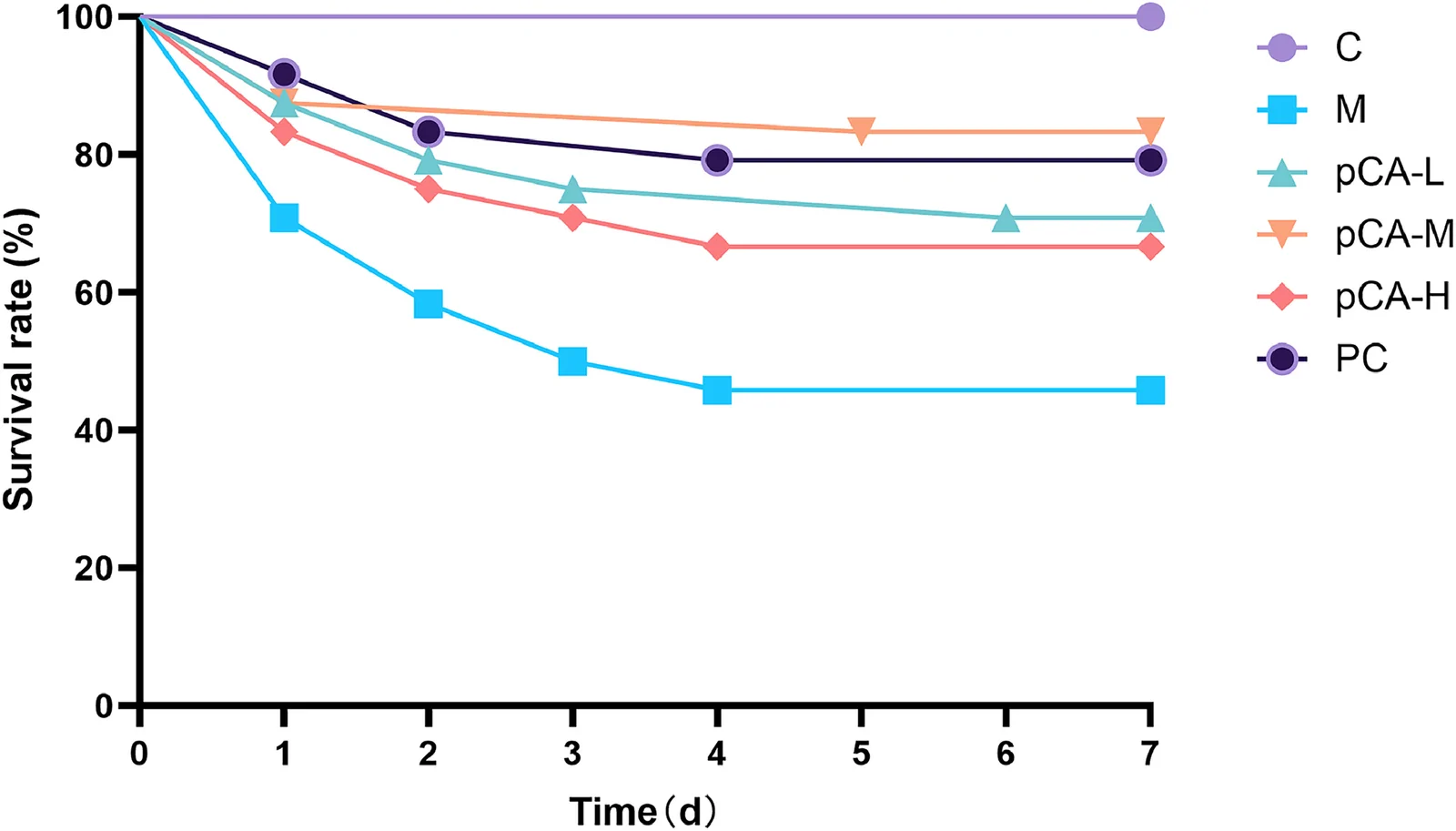
Effect of pCA on survival rate of chicks infected with APEC O2:K1. Curves represent: C, blank control; M, model; pCA-L, pCA 50 mg/kg; pCA-M, pCA 100 mg/kg; pCA-H, pCA 200 mg/kg; PC, enrofloxacin 100 mg/kg. Overall comparison among groups: log‑rank test, *P* < 0.001. Significant compared with group M: pCA‑M, *P* < 0.01; PC, *P* < 0.05. n = 24 per group.

During the 7‑d observation period, all chicks in the blank control group (group C) survived. The model group (group M) exhibited severe mortality, with a final survival rate of 45.83%. Overall survival curves differed significantly among the six groups (log‑rank test, *P* < 0.001; Fig. 6). Compared with group M, the survival rate was significantly improved in the pCA‑M group (100 mg/kg, 83.33%; *P* = 0.007) and in the positive control group (enrofloxacin 100 mg/kg, 79.16%; *P* = 0.016). The survival rates of the pCA‑L group (50 mg/kg, 70.83%) and the pCA‑H group (200 mg/kg, 66.67%) were numerically higher than that of group M, but the differences were not statistically significant. These results indicate that pCA improves the survival of infected chicks, with the medium dose (100 mg/kg) providing the optimal protective effect.

### Effect of pCA on organ lesions in infected chicks

**Fig. 7:**
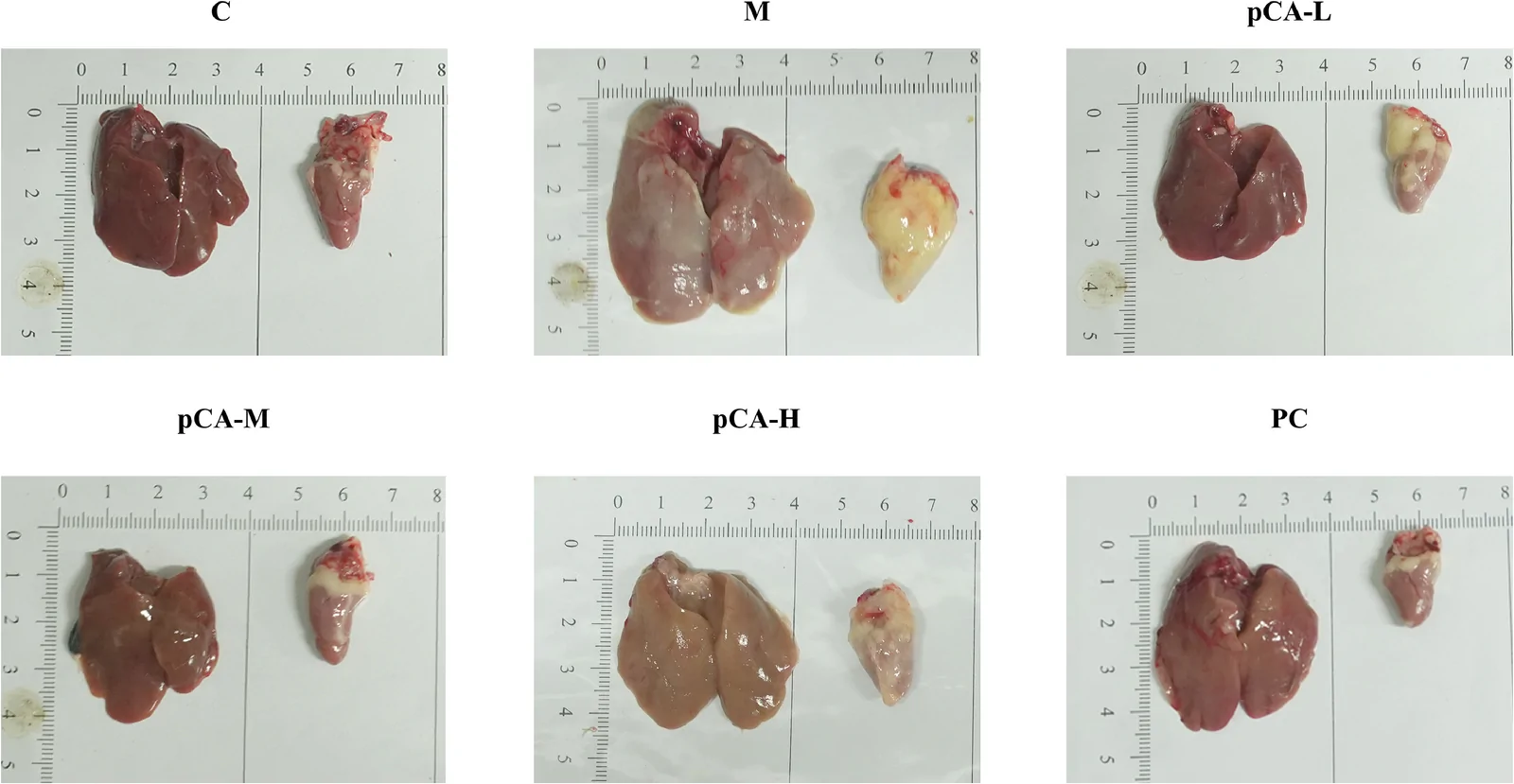
Effect of pCA on gross lesions of heart and liver in chicks infected with APEC O2:K1. Images show representative findings from six groups: (A) C, blank control; (B) M, model; (C) pCA-L, pCA 50 mg/kg; (D) pCA-M, pCA 100 mg/kg; (E) pCA-H, pCA 200 mg/kg; (F) PC, enrofloxacin 100 mg/kg.

Necropsy observations (Fig. 7) showed that chicks in group M exhibited typical pericarditis and perihepatitis: the heart was tightly wrapped by yellowish-white, thickened fibrinous exudate; the liver surface was covered with a dense grayish-white fibrinous membrane, with partial adhesion to the abdominal wall or pericardium. In the pCA-M group, heart and liver lesions were markedly alleviated. Most chicks in this group had heart and liver appearances that were largely restored to normal, with only mild pericarditis and perihepatitis observed in a few individuals. The PC group showed effects comparable to those of the pCA-M group. This indicates that pCA can alleviate organ damage caused by APEC.

### Effect of pCA on organ indices in infected chicks

As shown in Table 2, compared with group C, the heart, liver, lung, and spleen indices in group M were significantly increased (*P* < 0.001), indicating that APEC infection caused varying degrees of congestion, edema, and other pathological changes in various organs. Regarding immune organs, the thymus and bursa of Fabricius indices in group M were significantly decreased (*P* < 0.001), showing typical immune organ atrophy. Compared with group M, the pCA-M group showed significantly reduced heart, liver, lung, and spleen indices (*P* < 0.01 or *P* < 0.001), and significantly restored thymus and bursa of Fabricius indices (*P* < 0.001). The effect was comparable to that of the PC group. These results indicate that pCA effectively alleviates parenchymal organ damage caused by APEC infection and protects immune organs.

**Table 2:** Effect of pCA on organ indices of chicks infected with APEC O2:K1.

Group	C	M	pCA-L	pCA-M	pCA-H	PC
Heart index	10.51±1.35^⁎⁎⁎^	16.63±2.24	12.75±3.92*	11.62±3.15^⁎⁎⁎^	13.38±4.28	12.89±1.79^⁎⁎^
Liver index	32.50±6.143^⁎⁎⁎^	56.14±7.66	44.77±7.45*	38.04±7.82^⁎⁎⁎^	47.43±7.86	42.61±7.13^⁎⁎⁎^
Lung index	6.48±0.67^⁎⁎⁎^	8.09±1.02	7.07±0.57^⁎⁎^	6.43±0.63^⁎⁎⁎^	7.091±1.35^⁎⁎^	6.63±0.53^⁎⁎⁎^
Spleen index	1.14±0.33^⁎⁎⁎^	2.53±0.43	1.73±0.31^⁎⁎^	1.43±0.47^⁎⁎⁎^	1.82±0.26*	1.72±0.30^⁎⁎⁎^
Thymus index	1.73±0.54*	0.79±0.39	1.49±0.50	1.63±0.40*	1.40±0.59	1.61±0.56*
Bursa of Fabricius Index	2.64±0.31^⁎⁎⁎^	1.09±0.46	2.09±0.53	2.31±0.62^⁎⁎^	2.03±0.63	2.19±0.58^⁎⁎^

Note: Data are presented as mean ± SD (*n* = 10). *P < 0.05, **P < 0.01, ***P < 0.001 vs. model group.

### Effect of pCA on bacterial load in liver and blood of infected chicks

**Fig. 8:**
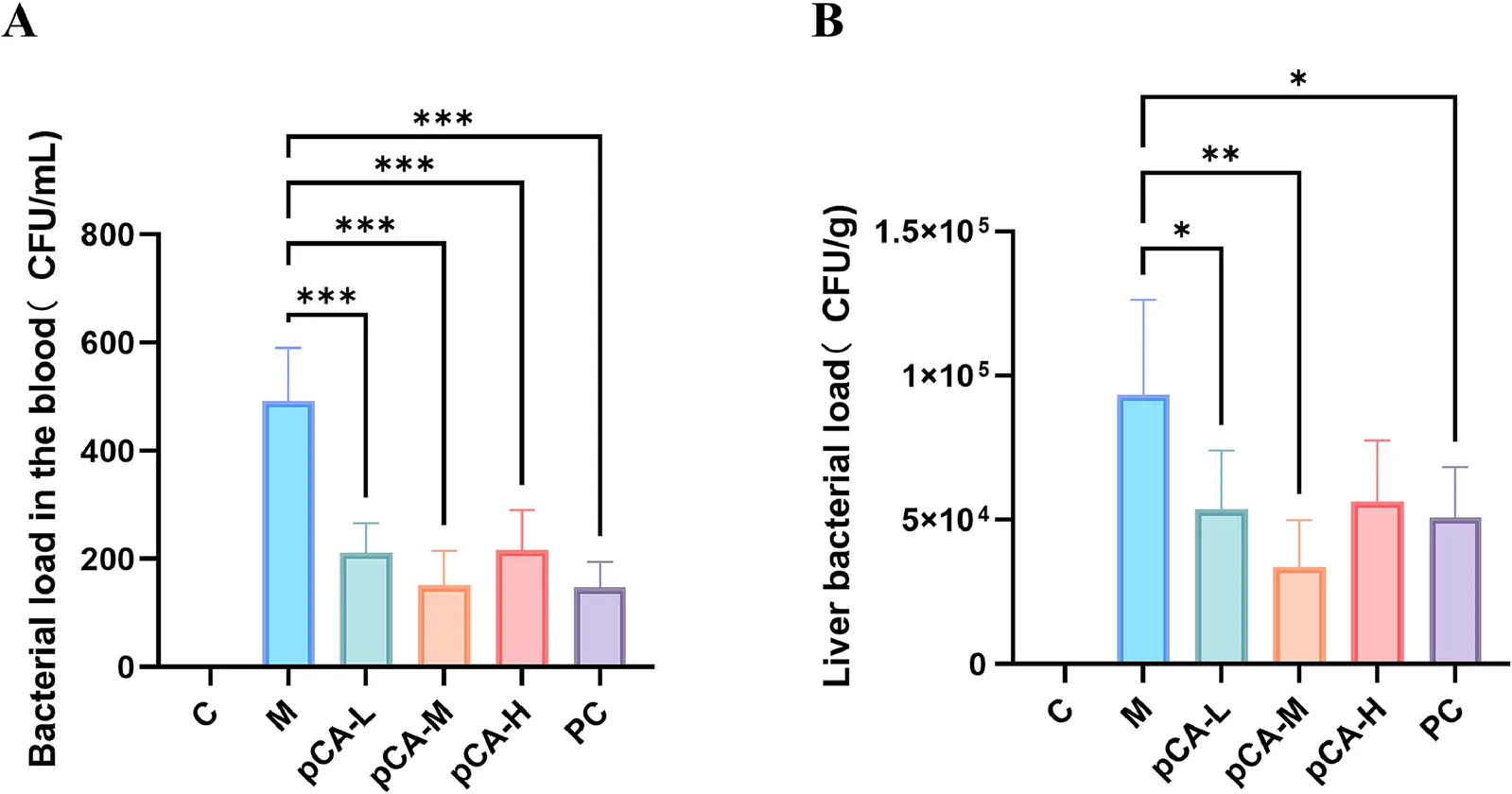
Effect of pCA on bacterial load in liver and blood of chicks infected with APEC O2:K1. (A) Liver bacterial load at 7 days post-infection. (B) Blood bacterial load at 7 days post-infection. Data are presented as mean ± SD (*n* = 10). *P < 0.05, ^⁎⁎^*P* < 0.01, ^⁎⁎⁎^*P* < 0.001 vs. model group.

The bacterial load results (Fig. 8) showed that compared with group M, all pCA treatment groups significantly reduced bacterial loads in the blood and liver of chicks (*P* < 0.05 or *P* < 0.01), with the pCA-M group showing the most pronounced clearance effect. These results indicate that pCA effectively clears colonized bacteria from infected chicks and inhibits bacterial dissemination in the blood and parenchymal organs.

### Effect of pCA on serum inflammatory cytokines, infection markers and immunoglobulins in infected chicks

**Fig. 9:**
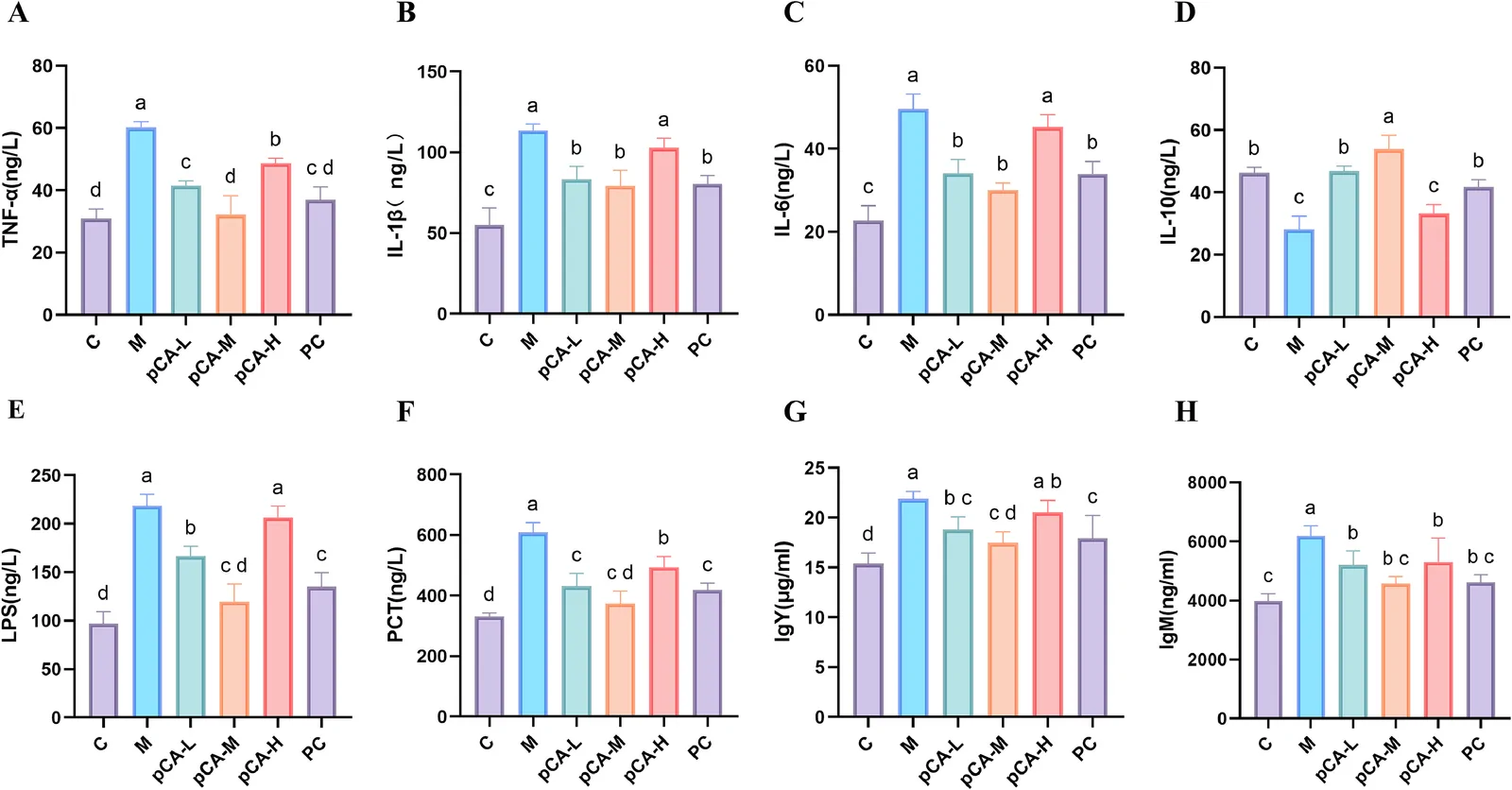
Effect of pCA on serum inflammatory cytokines, infection markers, and immunoglobulins in chicks infected with APEC O2:K1. Panels show: (A) TNF-α, (B) IL-1β, (C) IL-6, (D) IL-10, (E) LPS, and (F) PCT, (G) IgY, and (H) IgM. Data are presented as mean ± SD (*n* = 10). Different lowercase letters above bars indicate significant differences among groups (*P* < 0.05).

ELISA results (Fig. 9A - F) showed that compared with group C, serum levels of pro-inflammatory cytokines TNF-α, IL-1β, and IL-6 in group M were significantly elevated (*P* < 0.001), while the anti-inflammatory cytokine IL-10 level was significantly decreased (*P* < 0.05). Compared with group M, the pCA-M group showed significantly reduced levels of TNF-α, IL-1β, and IL-6 (*P* < 0.01 or *P* < 0.001), and a significantly increased level of IL-10 (*P* < 0.05). Regarding infection markers, serum LPS and PCT levels in group M were significantly elevated (*P* < 0.001), and the pCA-M group significantly reduced LPS and PCT levels (*P* < 0.05 or *P* < 0.01). These results indicate that pCA effectively suppresses the excessive inflammatory response induced by APEC infection and alleviates endotoxemia.

### Effect of pCA on serum immunoglobulins in infected chicks

As shown in Fig. 9(G - H), compared with group C, serum IgY and IgM levels in group M were significantly elevated (*P* < 0.001), indicating that infection activated the humoral immune response. Compared with group M, the pCA-M group significantly down-regulated IgY and IgM levels (*P* < 0.05), bringing them close to the levels of group C. These results indicate that pCA can regulate the humoral immune disorder caused by infection and promote the recovery of immune function.

### Effect of pCA on oxidative stress markers in serum and liver of infected chicks

**Fig. 10:**
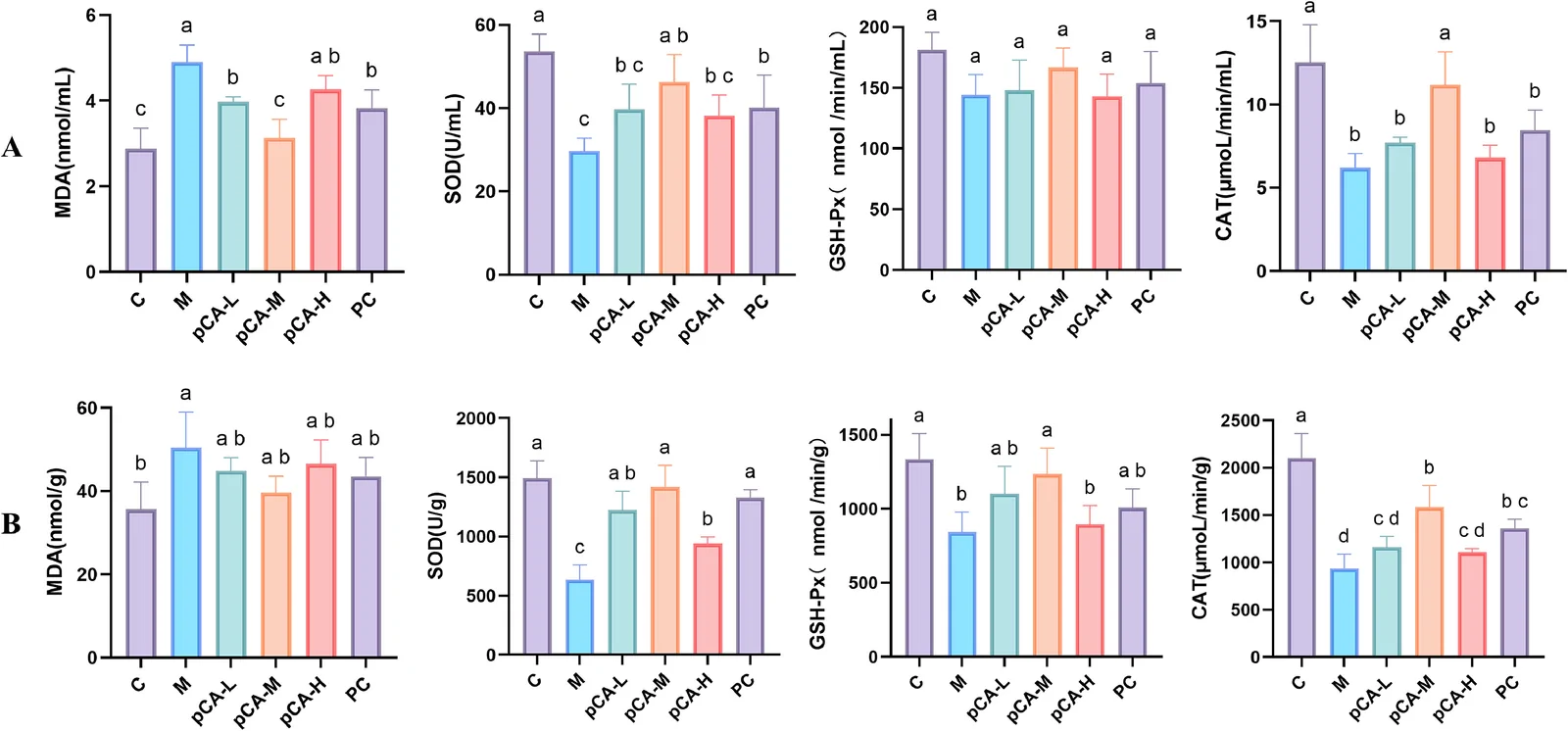
Effect of pCA on oxidative stress markers in serum and liver of chicks infected with APEC O2:K1. Panels show: (A) Serum MDA, SOD, GSH-Px activities and CAT content. (B) Liver MDA, SOD, GSH-Px activities and CAT content. Data are presented as mean ± SD (*n* = 10). Different lowercase letters above bars indicate significant differences among groups (*P* < 0.05).

The oxidative stress results (Fig. 10) showed that compared with group C, MDA content in serum and liver of group M was significantly increased (*P* < 0.001), while the activities of SOD, CAT, and GSH-Px were significantly decreased (*P* < 0.001), indicating that APEC infection caused severe oxidative stress damage. Compared with group M, the pCA-M group significantly increased the activities of SOD, CAT, and GSH-Px (*P* < 0.01) and significantly decreased MDA content (*P* < 0.01). The changes in liver indices were more pronounced than those in serum, which is consistent with the liver being the main target organ of APEC infection. These results indicate that pCA effectively ameliorates the oxidative stress state caused by APEC infection, enhances endogenous antioxidant enzyme activities, and reduces lipid peroxidation damage.

### Histopathological observations

**Fig. 11:**
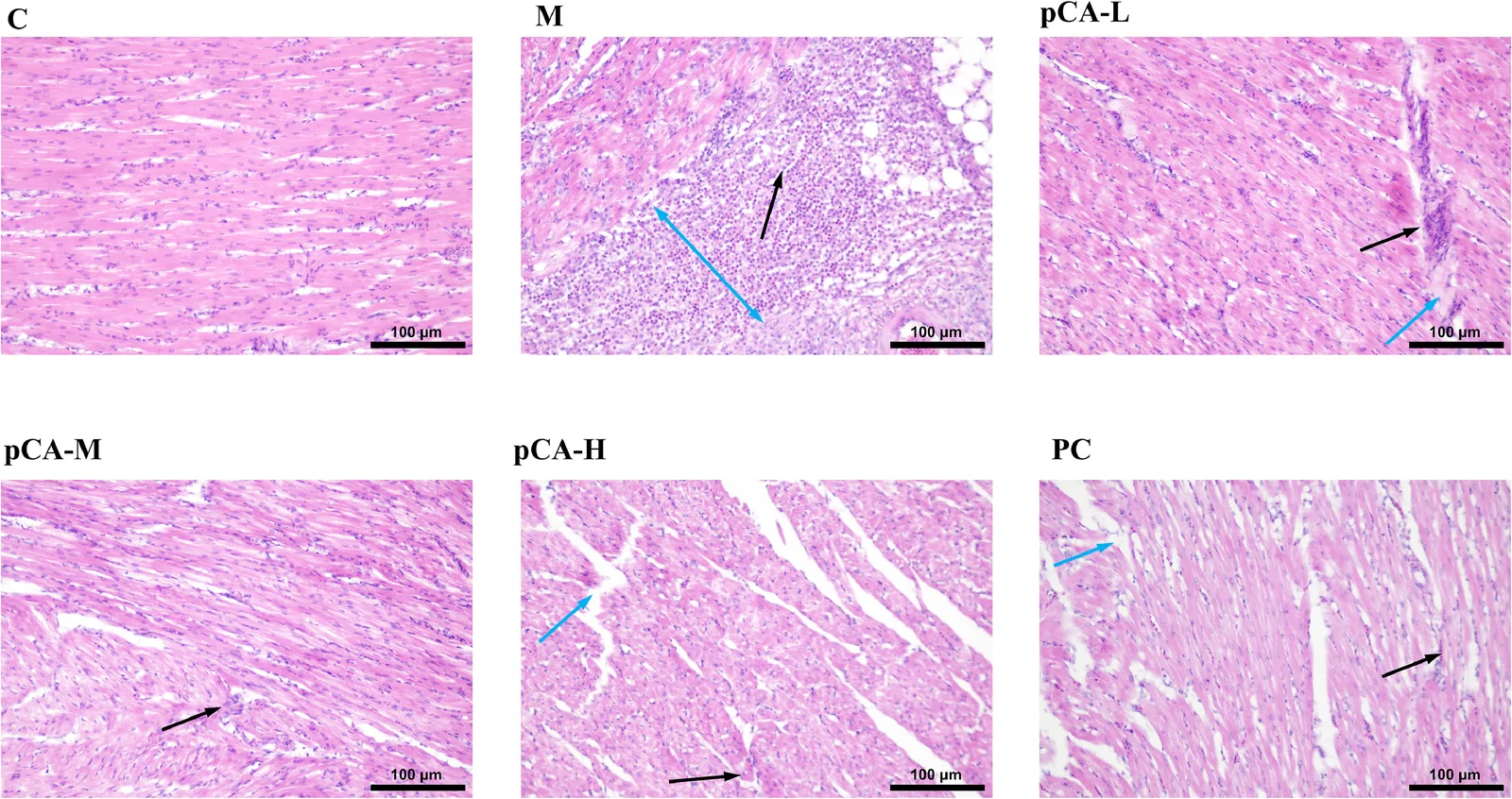
Effect of pCA on cardiac histopathology in chicks infected with APEC O2:K1 (HE, 200 ×). Black arrows indicate inflammatory cell infiltration; blue arrows indicate myocardial fiber edema, disordered arrangement, and structural damage. Scale bar = 100 μm.

HE staining of heart tissues (Fig. 11) showed that in group C, the myocardial tissue structure was intact, and myocardial fibers were arranged orderly and densely, with only a very small number of scattered physiological inflammatory cells. In group M, the myocardial tissue exhibited severe pathological damage, characterized by extensive diffuse inflammatory cell infiltration, widened intercellular spaces, edema, and rupture of myocardial fibers. In the pCA-M group, inflammatory infiltration in the myocardial tissue was significantly reduced, and the myocardial fiber structure was largely restored to normal, close to the level of group C.

**Fig. 12:**
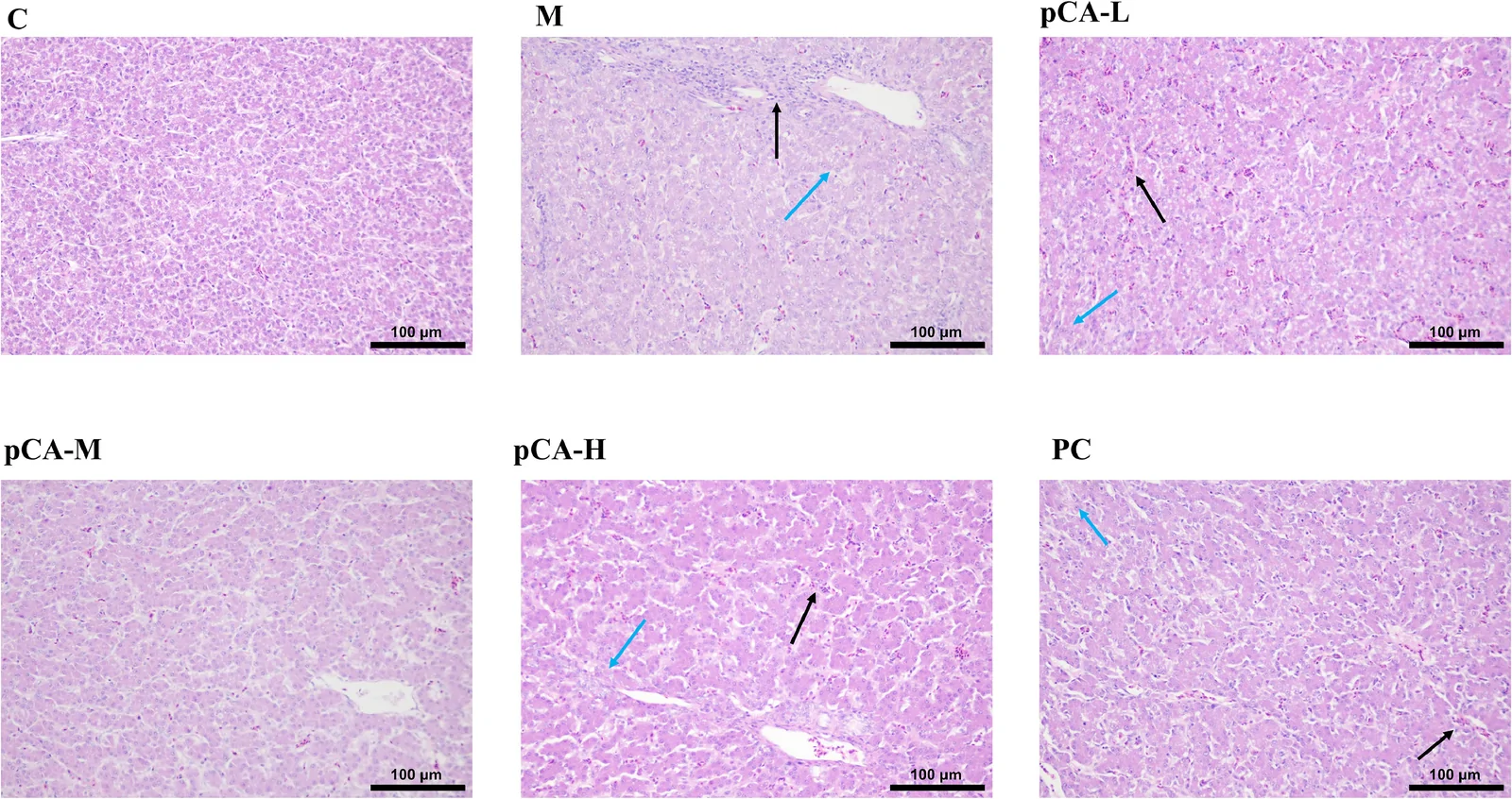
Effect of pCA on hepatic histopathology in chicks infected with APEC O2:K1 (HE, 200 ×). Black arrows indicate inflammatory cell infiltration; blue arrows indicate sinusoidal dilation and tissue edema. Scale bar = 100 μm.

HE staining of liver tissues (Fig. 12) showed that in group C, the liver tissue structure was intact, hepatic lobules were arranged regularly, hepatic cords were dense, and sinusoidal morphology was normal. In group M, the liver tissue exhibited severe pathological damage, characterized by extensive inflammatory cell infiltration, sinusoidal dilation, hepatocyte edema, vacuolar degeneration, and partial hepatocyte necrosis. In the pCA-M group, inflammatory infiltration in the liver tissue was significantly reduced, sinusoidal dilation was alleviated, and the hepatocyte structure was largely restored to normal. These results indicate that pCA effectively reverses the heart and liver tissue damage caused by APEC infection.

Semi-quantitative histopathological scoring confirmed these qualitative observations. The lesion scores for both heart and liver were significantly higher in the model group (heart: 2.75 ± 0.14; liver: 2.42 ± 0.15) compared to the blank control group (heart: 0.17 ± 0.11; liver: 0.00 ± 0.00) (*P* < 0.001). Treatment with pCA-M significantly reduced the lesion scores (heart: 0.83 ± 0.11; liver: 0.67 ± 0.14) compared to the model group (*P* < 0.01), consistent with the gross pathological findings.

## Discussion

The present study systematically evaluated the *in vitro* antibacterial activity and *in vivo* therapeutic efficacy of pCA against avian pathogenic *Escherichia coli* APEC O2:K1. In the *in vitro* experiment, the MIC of pCA against APEC O2:K1 was 3.125 mg/mL. The growth curve showed that bacterial growth was completely inhibited in the MIC group, while growth in the 1/2 MIC group began to recover after 7 h of culture, suggesting that bacteria may activate stress response mechanisms at sub-inhibitory concentrations (Amer et al., 2025). This phenomenon has clinical implications: when drug concentrations are insufficient to completely kill bacteria, bacteria may enter a tolerant state, increasing the risk of recurrent infection (Elawady et al., 2024). The cell membrane integrity assay showed that pCA treatment caused significant leakage of intracellular nucleic acids and proteins, and the leakage amount was positively correlated with drug concentration. This is consistent with the typical membrane-active characteristics of natural phenolic compounds (Liu et al., 2025). Studies have shown that phenolic acids possess membrane activity and can cause leakage of intracellular components (Borges et al., 2013). Ferulic acid, for example, disrupts the cell membrane integrity of pathogenic bacteria by altering hydrophobicity or causing localized membrane rupture (Liu et al., 2022), and pCA has also been demonstrated to have similar membrane-damaging effects (Li et al., 2022). The results of this study further confirm that pCA has the ability to directly disrupt the APEC cell membrane, which is the core basis for its immediate bactericidal effect. Biofilm formation is an important strategy for bacteria to resist antibiotics and host immune defense (Biswas et al., 2025). This study found that pCA inhibited APEC biofilm formation in a concentration-dependent manner. This is consistent with the inhibitory effect of pCA on uropathogenic *E. coli* biofilm reported by Kot et al. (Kot et al., 2015). The drug-containing plate diffusion assay further showed that pCA effectively inhibited bacterial spreading on solid medium. Bacterial spreading ability is closely related to flagellar motility and surface adhesion. Studies have confirmed that pCA can significantly inhibit bacterial motility and flagellar formation at sub-inhibitory concentrations by interfering with the quorum sensing system (Zhou et al., 2022). Together, these results suggest that pCA effectively weakens the colonization and spreading ability of APEC by inhibiting both biofilm formation and motility. Quorum sensing is an important communication system for bacteria to coordinate group behavior, regulating processes such as biofilm formation and virulence factor expression (Vadakkan et al., 2026). This study found that pCA at sub-inhibitory concentrations significantly down-regulated the expression of multiple key virulence genes in APEC. Among them, the expression of *luxS*, a key gene of the quorum sensing system, was significantly inhibited, suggesting that pCA may interfere with the bacterial quorum sensing regulatory network at the transcriptional level. This is consistent with the findings of Sun et al. (Sun et al., 2019) that berberine and matrine inhibit biofilm formation by down-regulating quorum sensing-related gene expression. Among adhesion and invasion-related genes, the expression of *fimH, tsh, ompA*, and *ibeA* decreased in a dose-dependent manner. The down-regulation of these genes directly impairs the colonization and invasion ability of APEC (Ovi et al., 2023). Regarding iron uptake and immune evasion, the expression of *iutA* and *iss* was significantly inhibited. Iron is an essential element for bacterial growth, and down-regulation of *iutA* affects the iron uptake capacity of APEC (Ibrahim et al., 2022), while down-regulation of *iss* enhances the clearance of APEC by host serum (Biran et al., 2021). In addition, the expression of *lpxC* and *pgsA* was also inhibited (van Beilen et al., 2016; Vinuesa et al., 2025), which corroborates the cell membrane damage observed in this study. Taken together, the in vitro results suggest that the anti-APEC effect of pCA is associated with three aspects: disruption of cell membrane integrity, inhibition of biofilm formation and bacterial spreading, and down-regulation of virulence gene expression.

Based on the *in vitro* findings, this study further evaluated the *in vivo* therapeutic efficacy of pCA using a chick APEC O2:K1 intraperitoneal infection model. The results showed that the 100 mg/kg pCA treatment group achieved a survival rate of 83.33%, which was significantly higher than that of the model group. This protective efficacy is superior to that reported by Meng et al. (Meng et al., 2024) for the combined treatment of matrine and berberine and the therapeutic effect of schisandrin A (Bao et al., 2021). Histopathological observations showed that after pCA treatment, myocardial fiber edema, rupture, and inflammatory infiltration were significantly reduced, and hepatocyte edema, vacuolar degeneration, sinusoidal dilation, and inflammatory cell infiltration were also improved. The organ index measurements were consistent with the pathological observations. pCA treatment significantly reduced the heart, liver, lung, and spleen indices, restored the thymus and bursa of Fabricius indices, and significantly cleared bacterial loads in blood and liver. Modern pharmacological studies have shown that natural plant monomers can alleviate organ damage caused by infection. For example, salvianolic acid B reduces myocardial injury in septic mice (Chen et al., 2024), and puerarin alleviates acetaminophen-induced liver injury (Zhou et al., 2023). The results of this study are consistent with these findings.

An intriguing finding of this study was the non - linear dose - response relationship, wherein the medium dose (100 mg/kg) consistently outperformed the high dose (200 mg/kg) across multiple parameters, including survival rate, organ indices, and bacterial clearance. This phenomenon is consistent with the hormetic (biphasic) dose-response characteristic commonly observed with polyphenolic compounds (Vrankova et al., 2026). Several mechanisms may account for this observation. First, at higher concentrations, pCA may exhibit reduced bioavailability due to saturation of intestinal absorption or increased metabolic clearance (Pei et al., 2016). Second, high-dose polyphenol supplementation may disrupt cellular redox balance, leading to pro-oxidant activity that partially counteracts its beneficial effects (Vrankova et al., 2026). Additionally, the pharmacokinetics and pharmacodynamics of hydroxycinnamic acids in poultry, including optimal dosing regimens, remain to be fully elucidated.

The systemic inflammation and oxidative stress triggered by APEC infection are core pathological mechanisms leading to multiple organ damage (Yu et al., 2024). In this study, pCA treatment significantly reduced serum levels of pro-inflammatory cytokines TNF-α, IL-1β, and IL-6, and increased the level of the anti-inflammatory cytokine IL-10, effectively correcting the inflammatory imbalance. Similarly, cinnamaldehyde reduces inflammatory cytokine levels in *E. coli* infection (Figueiredo et al., 2022); berberine inhibits LPS-induced inflammatory cytokine release (Li et al., 2022); and baicalin restores serum inflammatory levels in Mycoplasma gallisepticum infection by regulating the NLRP3 inflammasome and autophagy pathways (Ishfaq et al., 2021). Meanwhile, pCA significantly reduced serum LPS and PCT levels. LPS is a key endotoxin that triggers excessive inflammation (Riva et al., 2024), and the decrease in PCT directly reflects the control of systemic bacterial infection (Shoaib et al., 2024). These results indicate that pCA not only reduces bacterial counts but also effectively curbs the excessive host inflammatory response triggered by bacteria and their products. Immunoglobulin detection showed that serum IgY and IgM levels in the model group were significantly elevated, and pCA treatment restored them to near normal levels, indicating that pCA restores immune homeostasis by controlling infection. Studies have shown that stevia extract regulates serum immunoglobulin levels in laying hens (Wen et al., 2024), and baicalin reduces immune organ damage caused by M. gallisepticum infection through the Nrf2/HO-1 pathway (Li et al., 2019). Regarding oxidative stress, pCA significantly increased the activities of SOD, CAT, and GSH-Px in serum and liver of infected chicks, and reduced MDA content. The changes in liver indices were more pronounced than those in serum, which is consistent with the liver being the main target organ of APEC infection (Chenxi et al., 2022). The antioxidant activity of hydroxycinnamic acid derivatives originates from their phenolic hydroxyl structure, which directly scavenges free radicals and chelates pro-oxidant metal ions (López-Herrador et al., 2025). Chlorogenic acid, ferulic acid, and caffeic acid all exert antioxidant effects through similar mechanisms (Kikuzaki et al., 2002; Khan et al., 2016; Wei et al., 2025). pCA effectively alleviates the oxidative damage induced by APEC infection through this mechanism.

It should be noted that the intraperitoneal challenge model employed in this study, while widely used in APEC therapeutic evaluation studies (Bao et al., 2021; Meng et al., 2024), does not fully replicate the natural respiratory route of infection commonly observed in commercial poultry production. Nevertheless, this model provides a reproducible and controlled system for evaluating the systemic efficacy of pCA. Future studies utilising intratracheal or aerosol challenge models are warranted to further validate these findings.

Taken together, the *in vitro* results demonstrate that pCA achieves direct killing of APEC and attenuation of its pathogenic potential through disruption of cell membrane integrity, inhibition of biofilm formation and bacterial spreading, and down-regulation of virulence gene expression; *the in vivo* results demonstrate that pCA clears pathogens, suppresses excessive inflammatory responses, and enhances antioxidant defense capacity. The synergistic effect of direct antibacterial action and host protection constitutes the pharmacological basis for the anti-avian colibacillosis activity of pCA. That said, this study focused on a single APEC O2:K1 strain over a seven-day observation period, and broader clinical evaluation remains warranted.

## Conclusion

pCA exhibited an MIC of 3.125 mg/mL against APEC O2:K1. It inhibited bacterial growth, disrupted cell membrane integrity, suppressed biofilm formation and bacterial spreading, and down-regulated the expression of multiple virulence genes. Oral administration of pCA at 100 mg/kg significantly increased the survival rate of infected chicks, reduced organ damage, decreased bacterial loads, and improved inflammatory and oxidative stress markers. pCA exerts its anti-avian colibacillosis effects through direct antibacterial activity and regulation of host responses.

## Authors’ contributions

Yuxin Li: Conceptualization, Investigation, Writing – original draft; Baichang Xu: Data curation, Validation, Formal analysis; Yuxi Qin: Methodology, Software; Geyin Zhang: Formal analysis, Resources; Jia Cao: Supervision, Validation; Shiqi Luo: Investigation, Visualization; Jieyu Su: Data curation, Writing – review & editing; Yiming Li: Methodology, Validation; Zhongyang Ma: Resources, Supervision; Jiesheng Liao: Validation, Data curation, Writing – review & editing; Zhenqin Yan: Validation, Investigation, Writing – review & editing; Zhaojia Ding: Data curation, Validation, Writing – review & editing; Hongbin Si: Conceptualization, Funding acquisition, Project administration, Writing – review & editing.

## Authorship addition consent form

Manuscript ID: PSJ-d-26-01375

Manuscript Title: Inhibitory Effects of p-Coumaric Acid Against Avian Pathogenic Escherichia coli O2:K1

Journal: Poultry Science

## 1. Statement of adjustment

All original authors and three newly added co-authors of the above manuscript jointly issue this written consent to apply for updating the author list.

Three new co-authors to be added: Jiesheng Liao, Zhenqin Yan, Zhaojia Ding.

Their contributions fully meet the ICMJE authorship criteria and are specified as follows:

1. Jiesheng Liao: Methodology, Investigation, Validation
2. Zhenqin Yan: Investigation, Validation, Resources
3. Zhaojia Ding: Investigation, Data curation, Validation

The full author list and the Authors’ Contributions section in the revised manuscript have been updated to record the work completed by the three newly added authors.

## 2. Consent confirmation

1. All original authors have carefully read the complete revised manuscript, reviewed the updated author sequence, and unanimously agree to add Jiesheng Liao, Zhenqin Yan and Zhaojia Ding as co-authors without any dispute or objection.
2. The three newly added authors have fully read the revised manuscript, acknowledge their individual research contributions, and voluntarily consent to be listed as co-authors of this manuscript.
3. All authors confirm that there is no gift authorship, ghost authorship, undisclosed conflict of interest or any disagreement regarding authorship of this study.
4. We fully understand and accept the journal’s policy that no revisions to author list or institutional affiliations will be allowed after this revised manuscript is submitted. We promise that no further application for author modification will be submitted after this resubmission.

## 3. Signature List

Original Authors

1

## Conflict of interest statement

The authors of the manuscript entitled “Inhibitory Effects of p‑Coumaric Acid Against Avian Pathogenic Escherichia coli O2:K1 In Vitro and In Vivo” hereby declare that there are no conflicts of interest to disclose.

This work was supported by the National Key Research and Development Program of China (2023YFD1800100) and National Nature Foundation United Fund (U22A20523). The funders had no role in study design, data collection and analysis, decision to publish, or preparation of the manuscript. All authors have directly participated in the planning, execution, and analysis of this study. The authors have read and approved the final version submitted.

The materials within have not been and will not be submitted for publication elsewhere. There were no relationships with any entities that could be perceived to influence, or that give the appearance of potentially influencing, what was written in the submitted work.

The authors affirm that they have no financial affiliation (e.g., employment, direct payment, stock holdings, retainers, consultantships, patent licensing arrangements, or honoraria), or involvement with any commercial organization with direct financial interest in the subject or materials discussed in this manuscript, nor have any such arrangements existed in the past three years. Any other potential conflicts of interest have been disclosed to the best of our knowledge.

Hongbin Si

## Disclosures

The authors declare that there are no conflicts of interest.
